# Novel immunoprofiling method for diagnosing SLE and evaluating therapeutic response

**DOI:** 10.1136/lupus-2022-000693

**Published:** 2022-06-22

**Authors:** Jan-Mou Lee, Ming-Huang Chen, Kai-Yuan Chou, Yee Chao, Ming-Han Chen, Chang-Youh Tsai

**Affiliations:** 1 Department of Advanced Research, FullHope Biomedical Co Ltd, New Taipei City, Taiwan; 2 Faculty of Medicine, National Yang Ming Chiao Tung University, Taipei City, Taiwan; 3 Department of Oncology, Taipei Veterans General Hospital, Taipei, Taiwan; 4 Division of Immunology & Rheumatology, Fu Jen Catholic University Hospital, New Taipei City, Taiwan

**Keywords:** lupus erythematosus, systemic, autoimmunity, autoimmune diseases

## Abstract

**Objective:**

Diagnosis of SLE is based on clinical manifestations but is heterogeneous in early onset. Hence, we aimed to evaluate the feature of the immunoprofiling in patients with SLE and apply it to develop an immune signature algorithm for supporting SLE diagnosis.

**Methods:**

We enrolled 13 newly diagnosed patients with SLE and 9 healthy controls (HCs) followed by analysing their immunoprofilings within their peripheral blood mononuclear cells (PBMCs) through flow cytometry. The immunoprofiling from the patients with SLE and HCs were ranked and formed an immune signature score. Besides, we enrolled four patients with SLE and monitored the changes in their immunoprofilings after immunosuppressant treatment.

**Results:**

Among 93 immune cell subsets, 29 differed significantly between patients with SLE and HCs, and lower dendritic and natural killer cell percentages and a higher CD8^+^ T-cell percentage were identified in patients with SLE. In an investigation of immune-tolerant-related cell subsets, higher concentrations of CD8^+^ regulatory natural killer T cells, programmed cell death 1 (PD-1)^+^ T cells, and lower concentrations of programmed cell death ligand 1 (PD-L1)^+^ PBMCs were observed in the SLE group. The immune signature score from patients with SLE was significantly different from that from the HCs. After treatment, the disease activity of the four patients were tended to stable and percentages of PD-L1^+^ monocytes, PD-1^+^ CD4 T and CD8 T cells in patients with SLE exhibited positively and negatively correlation with the SLEDAI-2K (Systemic Lupus Erythematosus Disease Activity Index 2000) score, which might associate with the remission of SLE.

**Conclusions:**

The comparison of immunprofiling between patients with SLE and HCs exhibited a distinct pattern. This difference and its application to immune signature algorithm shed light on the studies of SLE pathogenesis and immune-based diagnostic tool development in the future.

**WHAT IS ALREADY KNOWN ON THIS TOPIC**

- Diagnostic tools based on the immunoprofilings of SLE are not available. No durable tool for assessing disease activity of SLE during treatment.

**WHAT THIS STUDY ADDS**

- We compared the immunoprofiling between patients with SLE and healthy controls and identified the alteration of regulatory and tolerant-related cells in patients with SLE. Ranking the change from these cells could accurately distinguish the immunoprofiling between patients with SLE and healthy controls. Also, the dynamic of cells associated with immune tolerance correlated with the clinical outcome after immunosuppressant treatment.

**HOW THIS STUDY MIGHT AFFECT RESEARCH, PRACTICE AND/OR POLICY**

- Immunoprofiling comparison and immune signature construction can support SLE diagnosis and research both bench-side and bed-side.

## Introduction

SLE is a severe autoimmune syndrome whose incidence in women is higher than in men.^1^ Via an unclear mechanism, patients with SLE lose their immune tolerance toward self-antigens (such as high-mobility group protein 1) so that autoreactive immune responses are stimulated.^2 3^ Common symptoms during SLE onset include systemic inflammation (such as fatigue, malar rash and fever), immune dysregulation (high levels of autoantibodies and low serum complement contents) and organ damage (such as nephritis, arthritis and peripheral neuropathy).^4^ Of note, diagnosis of SLE is challenging because the symptoms of SLE during early-onset can be non-specific and mimic other more common disorders.^5 6^

The content difference in immunoprofiling between patients with SLE and healthy controls (HCs) can characterise the dysregulation of the immune response.^7^ As compared with HCs, patients with SLE have lower percentages of the natural killer (NK) cell, dendritic cell (DC), regulatory cells and CD4^+^/CD8^+^ T-cell ratios (attributed to CD4 lymphocytopaenia) and higher percentages of B cells, double-negative T cells and regulatory CD4^+^ T cells.^8–14^ These alterations may be potential targets for monitoring the treatment efficacy of SLE disease activity.

The conventional diagnostic criteria for SLE rely on clinical manifestations and serum autoantibodies as indicators.^15^ The SLE Disease Activity Index (SLEDAI), Systemic Lupus Activity Measure Index and the British Isles Lupus Assessment Group Index (BILAG) are used to evaluate disease activity in patients with SLE in the clinic.^16–18^ However, these metrics may not be suitable for monitoring treatment efficacy because of dichotomous and subjective assessment criteria.^19 20^ Furthermore, no available diagnostic index for SLE disease activity based on immunoprofilings restricts the diagnostic accuracy. Accordingly, a novel and accurate diagnostic index for SLE management is essential. Patients with SLE and HCs exhibit a difference in their immunoprofilings. Our study aimed to comprehensively assess the differences in immunoprofilings between patients with SLE and HCs and determine the subsets featured in SLE. Furthermore, we used these feature subsets to construct a ranking algorithm that helps physicians in SLE diagnosis. Finally, we monitored the immunoprofilings of patients with SLE before and after immunosuppressant treatment to examine whether immunoprofilings are helpful for the precise clinical monitoring of disease activity.

## Materials and methods

### Participants and study design

We conducted an observational trial to compare the immunoprofiling between patients with SLE and the HCs in Taipei Veterans General Hospital (Taipei, Taiwan) with approval by their institutional review board. The study design is as follows. First, we compared the immunoprofiling between patients with SLE and HCs to characterise their immune signatures. Subsequently, patients with SLE received immunosuppressant therapy, and we determined their change of the immunoprofiling and clinical manifestation during the treatment. The eligible criteria of patients with SLE were newly diagnosed, ages between 20 and 65 years. The eligible criteria of the HCs were ages between 20 and 65 years with no medical history of inherited diseases, cancers, transplantation and were not pregnant. The physicians used the SLE classification criteria published by the European League Against Rheumatism (EULAR) and the American College of Rheumatism (ACR) in 2019, and SLEDAI-2K to determine the disease activity of the patients with SLE.^15 16^ A 20-mL sample of peripheral blood was collected at treatment started (week 0), during (week 4) and finished (week 12).

### Reagents and antibodies

Ficoll-Paque PREMIUM medium (density: 1.077 g/mL; Cytiva 17-5442-02, Marlborough, Massachusetts, USA) was applied to isolate human PBMC. Other general chemicals and reagents were purchased from Sigma-Aldrich (Merck, Darmstadt, Hessen, Germany).

We used 16 fluorescent-labelled antibodies from three manufacturers to label 15 specific cell markers and subsequently identify immune subsets. Allophycocyanin/Alexa Fluor 700 (APC/AF700)-conjugated CD56 (N901, B10822); APC/Alexa Fluor 750 (APC/AF750)-conjugated CD14 (RMO52, A86052) and CD19 (J3-119, A78838); Krome orange (KO)-conjugated CD3 (UCHT1, B00068) and CD8 (B9.11, B00067); pacific blue (PB)-conjugated HLA-DR (major histocompatibility complex class II (MHC II), immu-357, A74781); and phycoerythrin/cyanine 5.5 (PE/Cy5.5)-conjugated CD4 (SFCI12T4D11, 6607101) were obtained from Beckman-Coulter (Brea, California, USA). APC-conjugated CD11c (3.9, 301614) and T-cell receptor (TCR)γ/δ (236A/E7, 331212); fluorescein isothiocyanate-conjugated TCRα/β (L3D10, 306705); PB-conjugated CD69 (FN50, 310919); PE-conjugated CD25 (BC96, 302606), CTLA-4 (EH12.2H7, 349906) and PD-L1 (B1, 329706); and peridinin-chlorophyll-protein/Cy505 (PerCP/Cy5.5)-conjugated PD-1 (IP26, 329914) were obtained from BioLegend (San Diego, California, USA). APC-conjugated FoxP3 (29E.2A3, 17-4777-42) was purchased from ThermoFisher (Waltham, Massachusetts, USA). All antibodies were aliquoted as received and stored under recommended conditions until use.

### Immunostaining and analysis of PBMCs

Human PBMCs were isolated from peripheral blood using Ficoll density gradient centrifugation. Then, PBMCs were stained with antibodies under 4°C and in a dark environment for 30 min and aliquoted the stained PBMCs into two parts. One part of stained PBMCs were used to analyse their fluorescent patterns directly using flow cytometry (Navios, Beckman-Coulter), and another part of stained PBMCs were fixed and permeabilised by Foxp3/Transcription Factor Staining Buffer Set (eBioscience 00-5523-00, ThermoFisher) followed by anti-Foxp3 and anti-CTLA-4 antibody (diluted with staining buffer) staining. PBMCs with intracellular staining were undergone fluorescent analysis by flow cytometer.

### Data processing and immune signature calculation

We applied Kaluza software V.1.3 (Beckman-Coulter) in data collection from flow cytometry. Then, immune subsets from PBMCs were gated based on the definition given in online supplemental table 1. The content of each immune subset was represented by the percentage of the immune subset.

10.1136/lupus-2022-000693.supp1 Supplementary data

We represented the detailed process of immune signature construction in online supplemental figure 1. We applied all data in the construction of immune signature. The complete process of immune signature construction had three parts: data preprocessing, zero-zone determination and immune signature calculation. In the data preprocessing, the first quartile (Q1), third quartile (Q3) and IQR of each subset from the HCs were calculated.^21^ The outlier of an immune subset was determined, while the data were higher than Q3+1.5×IQR or smaller than Q1–1.5×IQR. Then, we eliminated the outliers and used the remains to calculate the average (AVG) and SD for zero-zone determination. To define the zero zone, we plotted a histogram with case numbers of subset versus AVG±(n×SD)/10 in each immune subset of the HC and SLE group and converted them into the cover ratio. We determined the cover ratio difference range-by-range using subtraction of cover ratios of the HC group to the SLE group and defined the particular range with the highest difference as the zero zones of such subset. To rank the content of subset members, we determined the decile of the zero-zone±(n×SD)/10 range and used that decile in ranking subset members with −1 to –2 and −3. Finally, we summarised the ranked number of each subset belonging to individual subjects into their immune signature.

10.1136/lupus-2022-000693.supp2 Supplementary data

### Statistical analysis

We applied an unpaired Student’s t-test to compare the difference of immune subsets pairwisely between the SLE and the HC group. GraphPad Prism V.5.0a (GraphPad Software, San Diego, California, USA) was used to create the histogram and perform statistical analysis. Immune subsets with statistical significance (p <0.05) were labelled.

## Results

### Patient characteristics

We enrolled 13 patients with SLE and 9 HCs in this study via invitation of the physician whose baseline description was in table 1. Among the 13 patients with SLE, 12 (92.3%) were women with a mean age of diagnosis of 45.9 years old. In the HCs, eight (88.9%) were women with a mean age of 38.3 years old. All (100%) patients with SLE exhibited high serum ANA titres (1:80), and 11 of 13 patients (84.6%) presented anti-double-stranded DNA antibodies.

**Table 1:** Demographics of enrolled subjects

Characteristics	SLE	HC	P value
	N=13	N=9	
Female, n (%)	12 (92.3)	8 (88.9)	1.000
Age at diagnosis of SLE, years, mean±SD	45.9±10.7	38.3±14.5	0.182
Immunological profiles			
ANA titre >1:80, n (%)	13 (100.0)	0 (0.0)	
Anti-SSA/Ro positive, n (%)	8 (61.5)	–	–
Anti-SSB/La positive, n (%)	2 (15.4)	–	–
Anti-Smith positive, n (%)	4 (30.8)	–	–
Anti-RNP positive, n (%)	4 (30.8)	–	–
Anti-dsDNA positive, n (%)	11 (84.6)	–	–
Clinical manifestations			
Haematological disorder, n (%)	11 (84.6)	–	–
Kidney involvement, n (%)	5 (38.5)	–	–
CNS involvement, n (%)	2 (15.4)	–	–
Psychosis, n (%)	1 (7.7)	–	–
Serositis, n (%)	2 (15.4)	–	–
Joint involvement, n (%)	10 (76.9)	–	–
Skin involvement, n (%)	11 (84.6)	–	–
SLEDAI-2K score, median (range)	8 (6–30)	–	–

CNS, central nervous system; dsDNA, double-stranded DNA; HC, healthy control; RNP, ribonucleoprotein; SLEDAI-2K, SLE Disease Activity Index 2000; SSA/Ro, Sjögren's-syndrome-related antigen A; SSB/La, lupus La protein.

### Lower DC, NK and cytotoxic T-cell responses in patients with SLE

**Figure 1:**
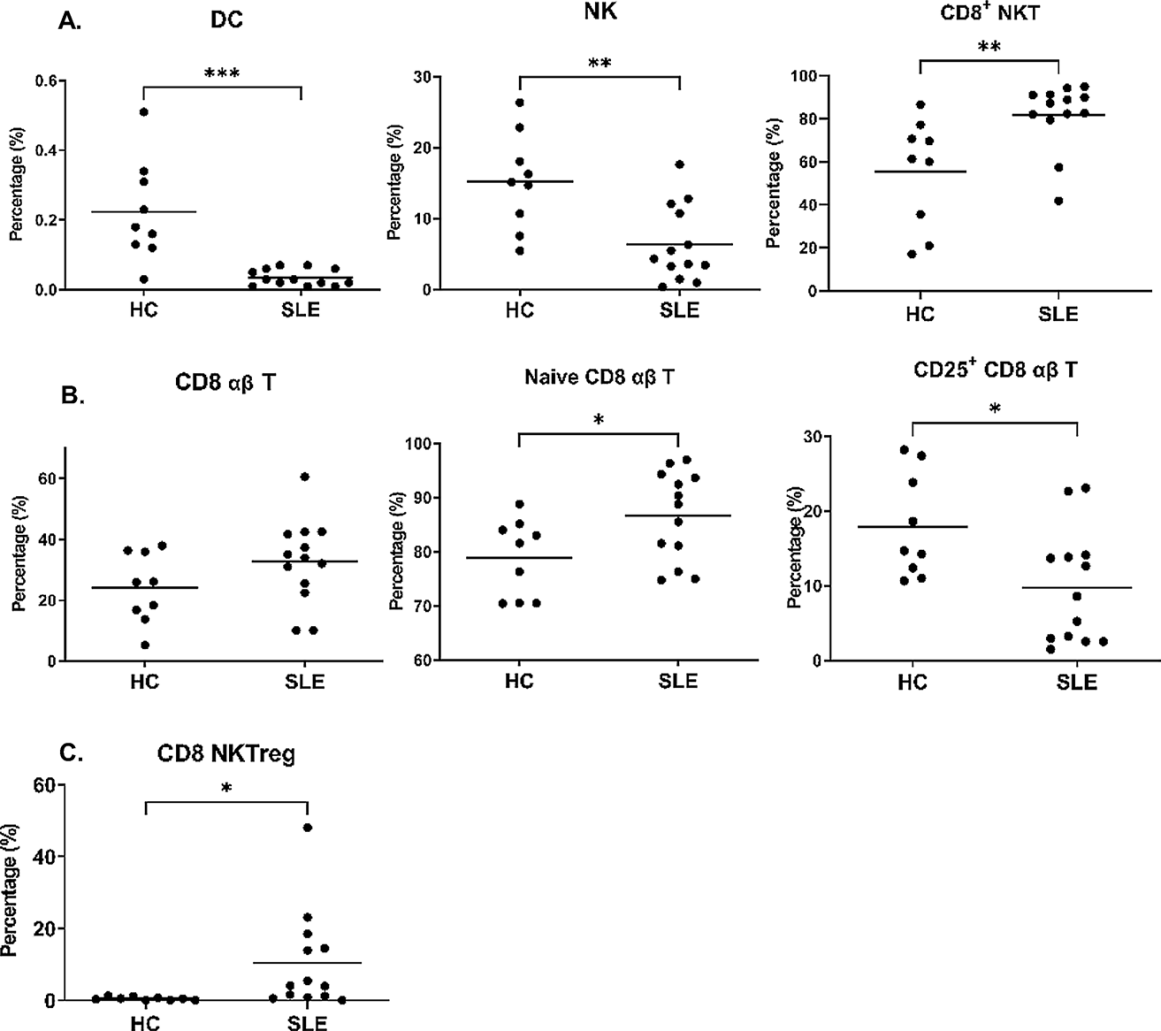
Patients with SLE carried distinct lineage-cell and T-cell patterns from those in the healthy controls (HCs). Peripheral blood mononuclear cells from the SLE group and the HC group were labelled with specific antibodies followed by grouping into lineage cell subsets (A), T cells (B) and regulatory cells (C) according to the subset definition enlisted in online supplemental table 1 by Kaluza and Prism. All data are shown in scatter plots with mean concentrations. DC, dendritic cell; NKT, natural killer T cell; Treg, regulatory T cell.

We analysed percentages of 93 immune cell subsets from PBMCs. About lineage cells, percentages of DCs and NKs in patients with SLE were significantly lower than those in the HCs (p=0.0001 and 0.0025, respectively; figure 1A). Percentages of CD8^+^ αβ T cells in patients with SLE were higher than those in the HCs (p=0.137; figure 1A). However, patients with SLE had higher percentages for naive CD8 αβ T cells and lower percentages for CD25^+^ CD8 αβ T cells relative to the HCs (p=0.0317 and 0.0183, respectively; figure 1B). According to previous studies, CD25 was a late activation marker of T cells and a characteristic marker of regulatory T (T_reg_) cells.^22 23^ No significant differences in percentages of CD8^+^ T_reg_ cells between the patients with SLE and the HCs (data not shown) indicated that the lower percentages of CD25^+^ CD8 αβ T cells in the SLE group were not attributed to a decrease in late activation of CD8 αβ T cells. Altogether, lower DC, NK and cytotoxic T-cell responses were observed in the patients with SLE than the HCs.

### Higher concentrations of regulatory CD8^+^ NKT cells in patients with SLE

In immune homeostasis, regulatory immune cells negatively moderate the immune response.^24^ Therefore, the dynamics of regulatory immune cells might participate in the pathogenesis of SLE.^25^ Notably, the higher percentages of CD8^+^ NKT cells were in the patients with SLE than those in the HCs (p=0.0061; figure 1A), which was attributable to higher percentages of CD8 NKT_reg_ cells (p=0.0427; figure 1C). The percentages of other regulatory immune cells, such as regulatory T cells and NKs, did not significantly differ between patients with SLE and HCs (data not shown). These results indicated slightly but significantly higher regulatory immune cell concentrations in the patients with SLE than the HCs.

### Imbalanced immune checkpoints in patients with SLE

**Figure 2:**
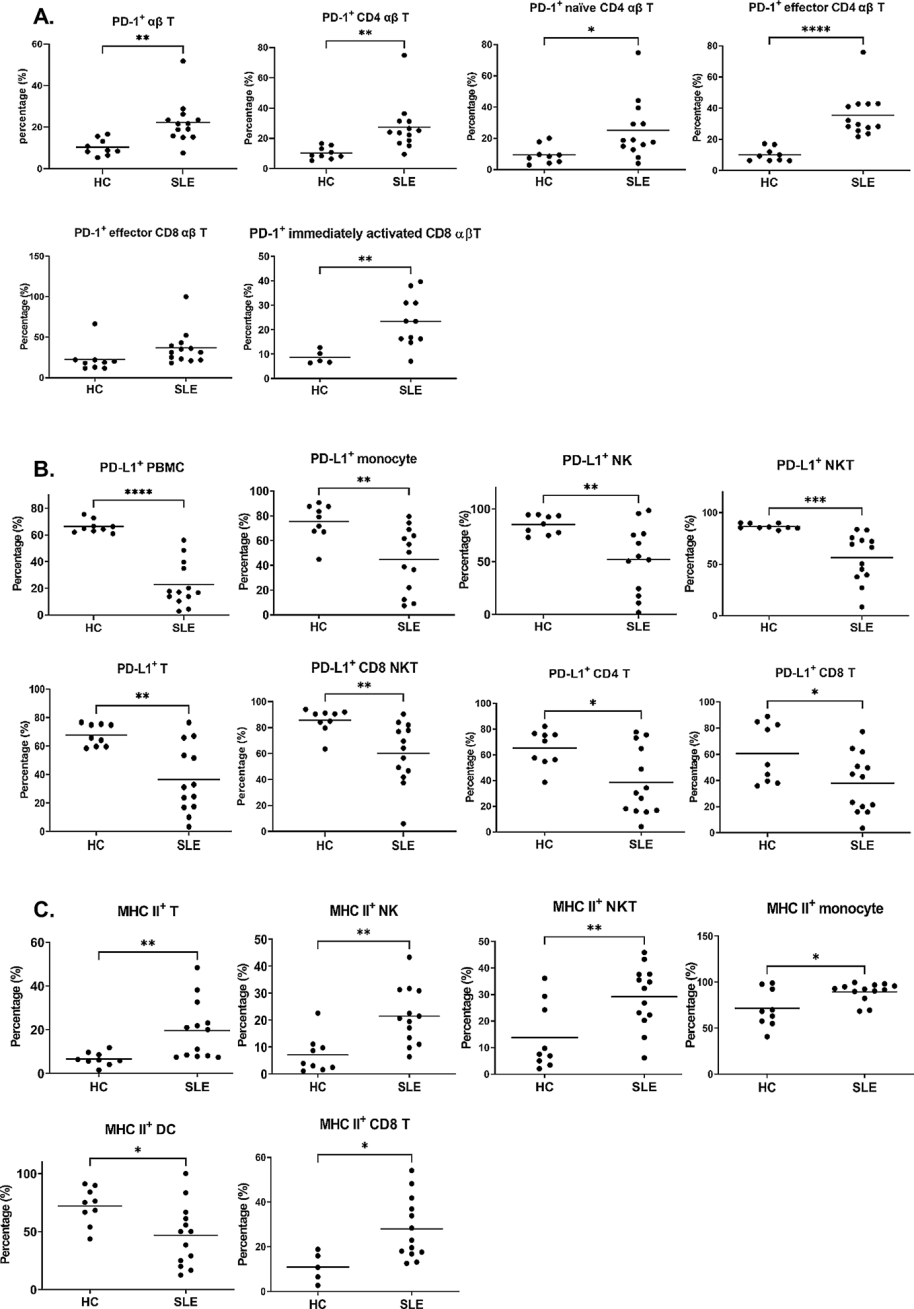
Patients with SLE carried dysregulated T-cell activation threshold. PBMCs were stained and filtrated with immune subsets via the above-described protocol. Immune subsets were grouped into PD-1^+^ cells (A), PD-L1^+^ cells (B) and MHC II^+^ cells (C) based on the definition of online supplemental table 1. Results are presented in scatter plots with the mean, and significant differences are labelled as follows: *p<0.05, **p<0.01 and ***p<0.001. DC, dendritic cell; HC, healthy control; MHC II, major histocompatibility complex class II; NKT, natural killer T cell; PBMCs, peripheral blood mononuclear cells; PD-1, programmed cell death 1; PD-L1, programmed cell death ligand 1.

Programmed cell death 1/programmed cell death ligand 1 (PD-1/PD-L1) interaction between immune cells modulated immune tolerance to self-antigens that inhibit activation of effector immune cells.^26^ The disruption of the PD-1/PD-L1 interaction participated in the pathogenesis of autoimmune diseases.^27^ Therefore, we measured the expression profiles of immune checkpoint proteins in PBMCs to assess self-tolerance degradation.^28^ As shown in figure 2A, higher percentages of PD-1^+^ αβ T cells were found in patients with SLE than those in the HCs (p=0.0044), which attributed to higher percentages of PD-1^+^ CD4 αβ T cells, PD-1^+^ naive CD4 αβ T and PD-1^+^CD25^+^ CD4 αβ T cells (figure 2A). In addition to PD-1^+^ CD4 αβ T cells, the percentages of PD-1^+^ effector CD8 αβ T cells and PD-1^+^ immediately activated CD8 αβ T cells were higher in the patients with SLE than those in the HCs (all p<0.05; figure 2A). Altogether, PD-1^+^ T-cell concentrations in patients with SLE were higher than those in HCs.

The percentages of PD-L1^+^ PBMCs were lower in the SLE group than in the HC group, which gave rise to a change in profile (figure 2B). The predominant subpopulations of PD-L1^+^ PBMCs, including PD-L1^+^ monocytes, NKs, NKT cells and T cells, were significantly lower in the SLE group (all p<0.05; figure 2B). The decreased percentages of PD-L1^+^ CD8 NKT cell were attributed to reduction of percentages of PD-L1^+^ NKT cells (figure 2B). For PD-L1^+^ T cells, both CD4^+^ αβ T and CD8^+^ αβ T cells exhibited lower percentages in the patients with SLE than those in the HCs (figure 2B). In conclusion, we identified overall higher PD-1^+^ and lower PD-L1^+^ immune cell percentages in the patients with SLE than those in the HCs, which may relate to the pathogenesis of SLE.

### Shift in the pattern of MHC II^+^ immune cells in patients with SLE

Studies had revealed that the polymorphism of the MHC II in immune cells altered the immune tolerance in which the presence of some MHC II alleles linked to autoimmune disease.^29^ However, the change of MHC II^+^ cell patterns in patients with SLE had not been investigated. Therefore, we compared the pattern of MHC II^+^ immune cells between the patients with SLE and the HCs. As displayed in figure 2C, higher percentages of MHC II^+^ NKs, NKT cells, monocytes, T cells and CD8 T cells were found in the patients with SLE than those in the HCs (all p<0.05; figure 2C). Percentages of MHC II^+^ DCs in the patients with SLE were significantly lower than those in the HCs (p<0.05; figure 2C). Collectively, augmented percentages of MHC II^+^ cells were identified in the patients with SLE than those in the HCs except DCs.

### Immune signature algorithm distinguishes patients with SLE from HCs

**Figure 3:**
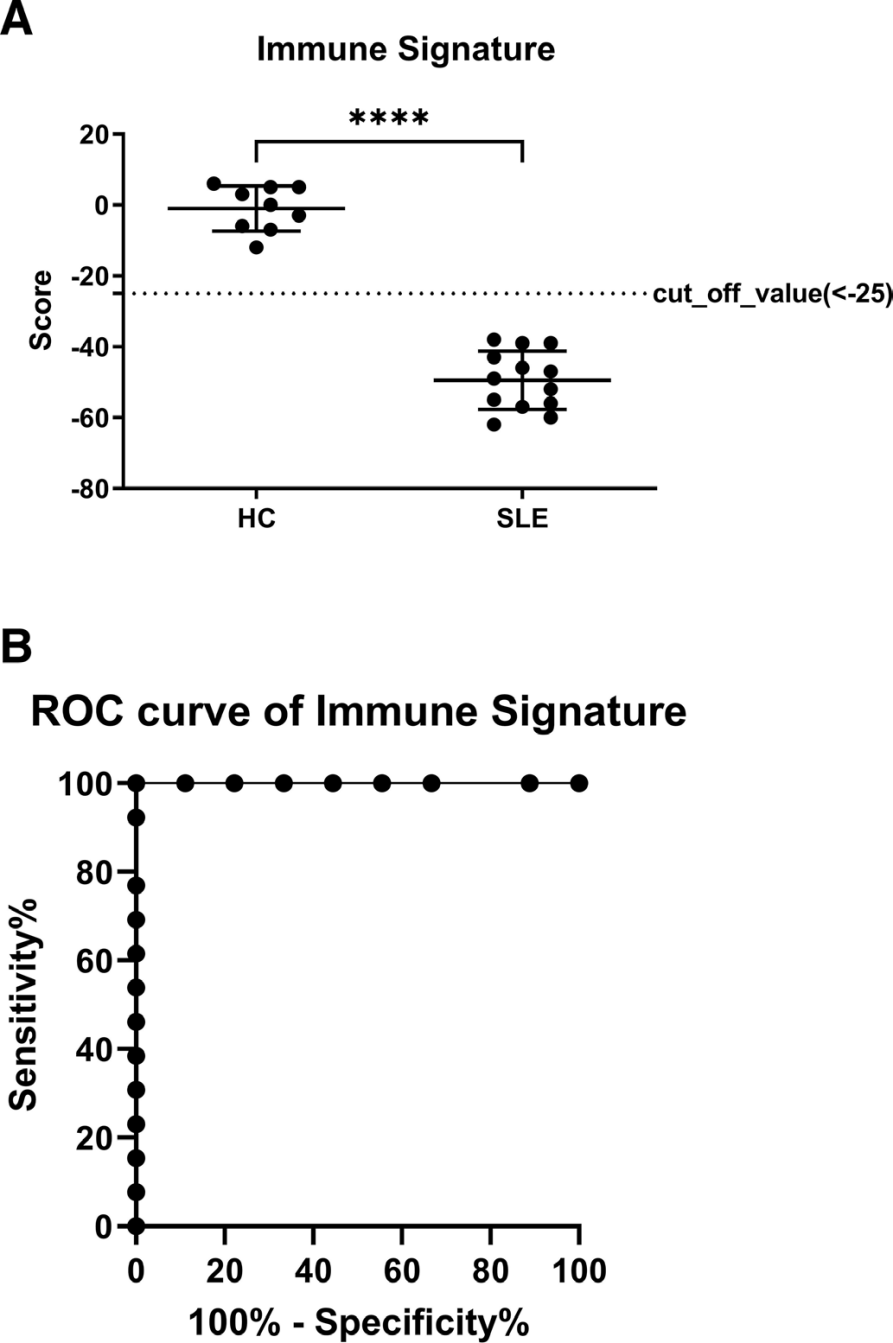
Immune signature of SLE and healthy control (HC) groups for comparison, and receiver operating characteristic (ROC) curve for the immune signature. The percentages of subsets from individual subjects proceeded to the ranking described in online supplemental figure 1 and summarised as the immune signature of the subject. The immune signature of the SLE and the HC group was compared (A). (B) An ROC curve was constructed to determine the performance of the immune signatures.

Subsequently, percentages of the immune cell subsets mentioned herein were ranked and summed to form an immune signature. The immune signature from the patients with SLE differed substantially from that of the HCs, for which the threshold was at −25 (figure 3A). We calculated the area under the curve (AUC) of the receiver operating characteristics to assess the performance of the immune signature. The AUC-based threshold for identifying patients with SLE with immune signature scores <25 was 1.00 (sensitivity=100.00%, specificity=100.00%, 95% CI 1.00 to 1.00, p<0.001; figure 3B). These results indicate that the immune signature could accurately distinguish patients with SLE from HCs.

### Immunosuppressant reversed PD-1/PD-L1 balance through an inversion in the pattern of PD-1/PD-L1 in patients with SLE

Finally, to monitor the dynamics of the immunoprofiling during immunosuppressant treatment, we determined the change of PD-1^+^ and PD-L1^+^ cells of four enrolled patients with SLE (clinical manifestation and regimen were in table 2) while they received immunosuppressant treatment. Four enrolled patients carried active lupus nephritis (three patients), alopecia (three patients), autoimmune haemolytic anaemia (two patients), mucosal ulcer (two patients) and low serum complement contents (two patients). Three of the four patients received methylprednisolone pulse therapy (1 g methylprednisolone for 3 consecutive days, once a month) followed by 50 mg of azathioprine or 200 mg of hydroxychloroquine, and the patient who did not carried active lupus nephritis received 5 mg of prednisolone once a day. After 12 weeks of treatment, the clinical outcome of the four patients tended to be stable.

**Table 2:** Clinical manifestation and regimens of monitored patients

Case ID	1	2	3	4
Clinical manifestation	Alopecia, mucosal ulcer, increased anti-dsDNA antibody, autoimmune haemolytic anaemia	Active lupus nephritis, psychosis, lupus headache, alopecia, increased anti-dsDNA antibody, low complement	Active lupus nephritis, low complement, autoimmune haemolytic anaemia	Active lupus nephritis, alopecia, mucosal ulcer, arthritis
**Treatment**				
MPT	No	Yes	Yes	Yes
Accompanied drug	No	No	CTX	No
Dose	No	No	450 mg, intravenous, every morning	No
**Following therapy**				
Drug	PRDL	AZA	HCQ	HCQ
Dose	5 mg orally, once a day	50 mg, intravenous, two times per day	200 mg, orally, four times per day	200 mg, orally, two times per day
Clinical outcome	Stable	Severe to stable	Severe to stable	Stable
2019 diagnostic criteria	16	29	24	21
SLEDAI-2K				
Week 0	6	30	10	12
Week 4	2	17	10	7
Week 12	2	17	10	10
Leucocyte count (×10^9^ counts/L)				
Week 0	7.0	14.4	3.8	7.5
Week 4	7.8	4.3	6.4	7.2
Week 12	7.4	17.4	5.4	4.0

The patients started receiving treatments (including methylprednisolone) after blood sample collection, and these treatments were naïve.

AZA, azathioprine; CTX, cyclophosphamide; dsDNA, double-stranded DNA; HCQ, hydroxychloroquine; MPT, methylprednisolone pulse therapy; PRDL, prednisolone; SLEDAI-2K, Systemic Lupus Erythematosus Disease Activity Index 2000.

**Figure 4:**
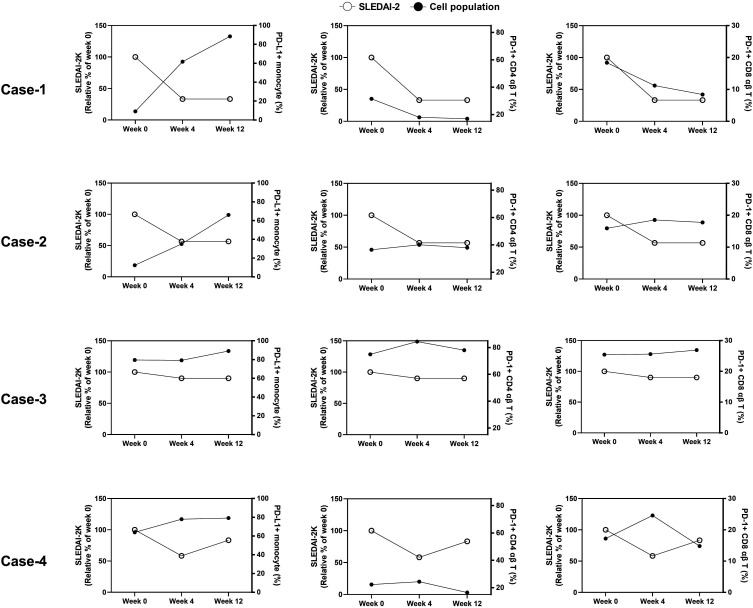
Positive and negative correlation of PD-L1^+^ cells and PD-1^+^ cells with disease activity from patient with SLE during immunosuppressant treatment. We enrolled four patients with SLE and monitored their disease activity (SLEDAI-2K and clinical manifestation), percentages of PD-1^+^CD4 T cells, PD-1^+^CD8 T cells and PD-L1^+^monocytes at the treatment started (week 0), week 4 and week 12. Clinical manifestations and treatment of the enrolled patients were described in table 2. The correlation between percentages of PD-1^+^CD4 T cells, PD-1^+^CD8 T cells and PD-L1^+^monocytes and SLEDAI-2K during the treatment was presented. PD-1, programmed cell death 1; PD-L1, programmed cell death ligand 1; SLEDAI-2K, Systemic Lupus Erythematosus Disease Activity Index 2000.

We measured the immunoprofiling and leucocyte counts of the four patients with SLE at the time point of weeks 0, 4 and 12. The leucocyte counts of case 1, 3 and 4 were around 4.0–7.8 ×10^9^ counts/L, which were in the normal range.^30^ Case 2 exhibited leucocytosis at the time point of week 0 (14.4 ×10^9^ counts/L) and week 12 (17.4 ×10^9^ counts/L). The longitudinal changes in the percentage of PD-L1^+^ monocytes, T cells, CD4 T cells and CD8 T cells were shown in figure 4. As a result, the dynamic trends of the SLEDAI-2K were negatively correlated with ones of PD-L1^+^ monocytes and positively correlated with ones of PD-1^+^ CD4 T and PD-1^+^ CD8 T cells. Although percentages of PD-L1^+^ monocytes in cases 3 and 4 were not significantly changed during the treatment, their percentages of PD-L1^+^ monocytes were at relatively high level. Similar trend could be observed in the dynamic trends of PD-1^+^ CD4 T and CD8 T cells. These results implied that augmentation of PD-L1^+^ monocyte and reduction of PD-1^+^ CD4 T and CD8 T cell might associate with the remission of SLE.

## Discussion

In this study, we compared the immunoprofiling of patients with SLE with the HCs and found lower percentages of peripheral blood DCs, NKs, PD-L1^+^ PBMCs, and MHC II^+^ DCs and higher of CD8^+^ NKT_reg_ cells, PD-1^+^ T cells, MHC II^+^ monocytes, NKs and NKT cells were found in patients with SLE. Subsequently, an immune signature algorithm based on the immunoprofiling from patients with SLE and HCs was constructed, which exhibited high sensitivity and specificity in distinguishing patients with SLE from HCs. In addition, after immunosuppressant treatment, PD-L1^+^ monocytes in patients with SLE tended to negatively correlate with the SLEDAI-2K scoring and the disease activity, which implied an association of the percentage of PD-L1^+^ monocytes with SLE remission.

The primary aim of this study was a comprehensive investigation of immunoprofilings in SLE. We observed lower percentages of DCs and NKs in the patients with SLE than those in the HCs and had reported in previous.^8 9^ Notably, our findings revealed that CD25^+^ CD8 αβ T-cell percentages were lower in the patients with SLE than those in the HCs even though the percentages of CD8^+^ αβ Treg cells were almost identical (figure 1B). CD25 is the inducible α chain of interleukin 2 (IL-2) receptor whose expression depends on autocrine or paracrine of IL-2 stimulation.^22^ That is, lower percentages of CD25^+^ CD8 αβ T cells may reflect the reduction of late activation of CD8 αβ T cells. Meanwhile, lower percentages of CD25^+^ CD8 αβ T cells in patients with SLE revealed the reduced secretion of IL-2, which was confirmed in the literature.^31^ Notably, we reported the novel phenomenon of higher percentages of CD8^+^ NKT cells in the patients with SLE than those in the HCs for the first time. Characteristics of CD8^+^ NKT cells in SLE development are contradictory because they participate in SLE progression via producing interferon-γ.^32–34^ However, CD8^+^ NKT cells reduce antigen-bearing DCs so that T-cell response and development of autoimmune diseases are attenuated.^35–37^ Hence, the pathological role of CD8^+^ NKT cells in SLE development is unclear and needs more investigation to unveil.

After investigating the profile of PD-1^+^/PD-L1^+^ cells, we identified higher percentages of PD-1^+^ T cells and lower percentages of PD-L1^+^ PBMCs in patients with SLE than HCs (figure 2A, B). PD-L1 on somatic cells interacts with PD-1 on the cytotoxic T cells and inactivates them.^38^ PD-1/PD-L1 dysregulation involves the pathogenesis of autoimmune diseases, including rheumatoid arthritis and multiple sclerosis.^39 40^ In SLE, higher percentages of PD-1^+^CD4^+^ T cells, PD-1^+^TIM-3^+^ NKs and PD-1^+^ follicular helper-like T cells are identified than in the HCs which may attribute to accumulation of autoreactive T cells.^41–44^ Our results revealed higher percentages of PD-1^+^ αβ T cells in the patients with SLE than those in the HCs (figure 2A). Moreover, reduced percentages of PD-L1^+^ PBMCs attributed to PD-L1^+^ T cells, NKs, monocytes and NKT cells in the patients with SLE than those in the HCs. Reversed PD-1/PD-L1 trend may modulate PD-1 signalling and cause dysregulation of T-cell response in SLE.^45^

In addition to identifying PD-1^+^/PD-L1^+^ T-cell interaction, we identified decreased percentages of PD-L1^+^ NKs, monocytes and NKT cells in patients with SLE compared with the HCs (figure 2B). PD-L1^+^ monocytes and PD-L1^+^ NKs participate in immune tolerance via retraining antigen-specific T cells.^46 47^ The characteristic of PD-L1^+^ NKT cells in SLE progression is still unknown. Nevertheless, NKT cells contain both NK cell and T-cell activities.^48^ The potential role of PD-L1^+^ NKT cells might be similar to T cells and NKs. Depleting PD-1-specific lymphocytes on non-obese diabetic mice attenuates symptoms of autoimmune encephalomyelitis on them without a negative effect on the adaptive immune response.^49^ In the current study, percentages of PD-L1^+^ monocytes in patients with SLE were tended to inversely correlate with the score of SLEDAI-2K (figure 4). Inversely, percentages of PD-1^+^CD4 T and CD8 T cells were tended to positively correlate with the score of SLEDAI-2K. In comparison of PD-1/PD-L1 patterns between the patients with SLE and the HCs, augmented percentages of PD-1^+^ CD4 αβ T cells and reduced percentages of PD-L1^+^ monocytes were identified (figure 2A, B). These results implied that augmentation of PD-L1^+^ monocytes and reduction of PD-1^+^ T cells in patients with SLE might correlate to the remission of SLE.

Monitoring disease activity is still challenging in SLE. The conventional tools for evaluating disease activity in SLE, such as the SLEDAI-2K or BILAG-2004, are based on clinical manifestations and laboratory findings.^16 17^ However, the SLEDAI-2K is insensitive to symptom amelioration due to the similarity of its scoring criteria with that of the 2019 EULAR/ACR criteria.^50^ Therefore, although research suggests that these diagnostic instruments may reflect or even anticipate patient response to immunosuppressive therapy, a diagnostic tool based on immune cell profiles is still lacking. The immune signature score we established functions independently of the presence of clinical manifestations and can provide a detailed picture of immune cell dynamics during treatment.

The limitations of this study were its small sample size. For future studies, larger-scale investigations of the use of immunoprofilings in the refinement of immune signatures are essential.

## Conclusion

In conclusion, we successfully characterised the immune signatures of patients with SLE and the dynamics of immunoprofilings after immunosuppressant therapy; these can serve as an indicator of disease activity and thus aid in the diagnosis of SLE.

## Data Availability

No data are available.
