# Supplementary material for: Novel immunoprofiling method for diagnosing SLE and evaluating therapeutic response
Source: Lupus Sci Med. 2022 Jun 22;9(1):e000693. doi: 10.1136/lupus-2022-000693 (PMC9226994; doi:10.1136/lupus-2022-000693)
Supplement: Supplementary data [file lupus-2022-000693supp001.pdf]

Supplementary Table 1. Definition of immune cell subsets with markers

Cell name	Markers
Lineage cell	
Monocyte	$CD3^-CD14^+CD19^-CD56^-$
NK	$CD3^-CD14^-CD19^-CD56^+$
CD4 NK	$CD3^-CD14^-CD19^-CD56^+CD4^+CD8^-$
CD8 NK	$CD3^-CD14^-CD19^-CD56^+CD4^-CD8^+$
NKT	$CD3^+CD14^-CD19^-CD56^+$
DC	$CD3^-CD14^-CD19^-CD56^-CD11c^+$
B	$CD3^-CD14^-CD19^+CD56^-$
T lymphocyte	
Total T	$CD3^+CD14^-CD19^-CD56^-$
αβ T	$CD3^+CD14^-CD19^-CD56^-TCR_{\alpha\beta}^+TCR_{\gamma\delta}^-$
γδ T	$CD3^+CD14^-CD19^-CD56^-TCR_{\alpha\beta}^-TCR_{\gamma\delta}^+$
CD4 αβ T	$CD3^+CD14^-CD19^-CD56^-TCR_{\alpha\beta}^+TCR_{\gamma\delta}^-CD4^+CD8^-$
CD8 αβ T	$CD3^+CD14^-CD19^-CD56^-TCR_{\alpha\beta}^+TCR_{\gamma\delta}^-CD4^-CD8^+$
Naïve CD4 αβ T	$CD3^+CD14^-CD19^-CD56^-TCR_{\alpha\beta}^+TCR_{\gamma\delta}^-CD4^+CD8^-$ $CD25^-CD69^-$
Naïve CD8 αβ T	$CD3^+CD14^-CD19^-CD56^-TCR_{\alpha\beta}^+TCR_{\gamma\delta}^-CD4^-CD8^+$ $CD25^-CD69^-$
$CD25^+$ CD4 αβ T	$CD3^+CD14^-CD19^-CD56^-TCR_{\alpha\beta}^+TCR_{\gamma\delta}^-CD4^+CD8^-$ $CD25^+CD69^-$
$CD25^+$ CD8 αβ T	$CD3^+CD14^-CD19^-CD56^-TCR_{\alpha\beta}^+TCR_{\gamma\delta}^-CD4^-CD8^+$ $CD25^+CD69^-$
Effector CD4 αβ T	$CD3^+CD14^-CD19^-CD56^-TCR_{\alpha\beta}^+TCR_{\gamma\delta}^-CD4^+CD8^-$ $CD25^-CD69^+$
Effector CD8 αβ T	$CD3^+CD14^-CD19^-CD56^-TCR_{\alpha\beta}^+TCR_{\gamma\delta}^-CD4^-CD8^+$ $CD25^-CD69^+$
Immediately activated CD4 αβ T	$CD3^+CD14^-CD19^-CD56^-TCR_{\alpha\beta}^+TCR_{\gamma\delta}^-CD4^+CD8^-$ $CD27^-CD28^-$
Immediately activated CD8 αβ T	$CD3^+CD14^-CD19^-CD56^-TCR_{\alpha\beta}^+TCR_{\gamma\delta}^-CD4^-CD8^+$ $CD27^-CD28^-$
Resting CD4 αβ T	$CD3^+CD14^-CD19^-CD56^-TCR_{\alpha\beta}^+TCR_{\gamma\delta}^-CD4^+CD8^-$ $CD27^+CD28^+$
Resting CD8 αβ T	$CD3^+CD14^-CD19^-CD56^-TCR_{\alpha\beta}^+TCR_{\gamma\delta}^-CD4^-CD8^+$ $CD27^+CD28^+$
Regulatory cells	
CD4 NKreg	$CD3^-CD14^-CD19^-CD56^+CD4^+CD8^-FoxP3^+CD25^+$

CD8 NKreg	$CD3^-CD14^-CD19^-CD56^+CD4^-CD8^+FoxP3^+CD25^+$
CD4 NKTreg	$CD3^+CD14^-CD19^-CD56^+CD4^+CD8^-FoxP3^+CD25^+$
CD8 NKTreg	$CD3^+CD14^-CD19^-CD56^+CD4^-CD8^+FoxP3^+CD25^+$
CD4 Treg	$CD3^+CD14^-CD19^-CD56^-CD4^+CD8^-FoxP3^+CD25^+$
CD8 Treg	$CD3^+CD14^-CD19^-CD56^-CD4^-CD8^+FoxP3^+CD25^+$
MDSC	$CD3^-CD14^+CD19^-CD56^-CD11b^+HLA\text{-}DR^-$
Myeloid cell	$CD3^-CD14^-CD19^-CD56^-CD11b^+HLA\text{-}DR^-$
AI-MC-1	$CD3^-CD14^-CD19^-CD56^-CD11b^+HLA\text{-}DR^-CD33^+CD39^+CD13^{hi}CD11c^+$
AI-MC-2	$CD3^-CD14^-CD19^-CD56^-CD11b^+HLA\text{-}DR^-CD33^+CD39^-CD13^{dim}CD11c^+$
PD-1⁺ cells	
$PD\text{-}1^+$ PBMC	$PD\text{-}1^+PD\text{-}L1^-$
$PD\text{-}1^+$ monocyte	$CD3^-CD14^+CD19^-CD56^-PD\text{-}1^+PD\text{-}L1^-$
$PD\text{-}1^+$ NK	$CD3^-CD14^-CD19^-CD56^+PD\text{-}1^+PD\text{-}L1^-$
$PD\text{-}1^+$ NKT	$CD3^+CD14^-CD19^-CD56^+PD\text{-}1^+PD\text{-}L1^-$
$PD\text{-}1^+$ CD4 NKT	$CD3^+CD14^-CD19^-CD56^+CD4^+CD8^-PD\text{-}1^+PD\text{-}L1^-$
$PD\text{-}1^+$ CD8 NKT	$CD3^+CD14^-CD19^-CD56^+CD4^-CD8^+PD\text{-}1^+PD\text{-}L1^-$
$PD\text{-}1^+$ DC	$CD3^-CD14^-CD19^-CD56^-CD11c^+PD\text{-}1^+PD\text{-}L1^-$
$PD\text{-}1^+$ αβ T	$CD3^+CD14^-CD19^-CD56^-TCR_{\alpha\beta}^+TCR_{\gamma\delta}^-PD\text{-}1^+PD\text{-}L1^-$
$PD\text{-}1^+$ γδ T	$CD3^+CD14^-CD19^-CD56^-TCR_{\alpha\beta}^-TCR_{\gamma\delta}^+PD\text{-}1^+PD\text{-}L1^-$
$PD\text{-}1^+$ CD4 T	$CD3^+CD14^-CD19^-CD56^-CD4^+CD8^-PD\text{-}1^+PD\text{-}L1^-$
$PD\text{-}1^+$ CD8 T	$CD3^+CD14^-CD19^-CD56^-CD4^-CD8^+PD\text{-}1^+PD\text{-}L1^-$
$PD\text{-}1^+$ naive CD4 T	$CD3^+CD14^-CD19^-CD56^-CD4^+CD8^-CD25^-CD69^-PD\text{-}1^+PD\text{-}L1^-$
$PD\text{-}1^+$ naive CD8 T	$CD3^+CD14^-CD19^-CD56^-CD4^-CD8^+CD25^-CD69^-PD\text{-}1^+PD\text{-}L1^-$
$PD\text{-}1^+$ $CD25^+$ CD4 T	$CD3^+CD14^-CD19^-CD56^-CD4^+CD8^-CD25^+CD69^-PD\text{-}1^+PD\text{-}L1^-$
$PD\text{-}1^+$ $CD25^+$ CD8 T	$CD3^+CD14^-CD19^-CD56^-CD4^-CD8^+CD25^+CD69^-PD\text{-}1^+PD\text{-}L1^-$
$PD\text{-}1^+$ effector CD4 T	$CD3^+CD14^-CD19^-CD56^-CD4^+CD8^-CD25^-CD69^+PD\text{-}1^+PD\text{-}L1^-$
$PD\text{-}1^+$ effector CD8 T	$CD3^+CD14^-CD19^-CD56^-CD4^-CD8^+CD25^-CD69^+PD\text{-}1^+PD\text{-}L1^-$
$PD\text{-}1^+$ Immediately activated CD4 αβ T	$CD3^+CD14^-CD19^-CD56^-TCR_{\alpha\beta}^+TCR_{\gamma\delta}^-CD4^+CD8^-CD27^-CD28^-PD\text{-}1^+PD\text{-}L1^-$

PD-1^{+} Immediately activated CD8 αβ T	CD3^{+}CD14^{-}CD19^{-}CD56^{-}TCR$_{αβ}^{+}$TCR$_{γδ}^{-}$CD4^{-}CD8^{+} CD27^{-}CD28^{-}PD-1^{+}PD-L1^{-}
PD-1^{+} Resting CD4 αβ T	CD3^{+}CD14^{-}CD19^{-}CD56^{-}TCR$_{αβ}^{+}$TCR$_{γδ}^{-}$CD4^{+}CD8^{-} CD27^{+}CD28^{+}PD-1^{+}PD-L1^{-}
PD-1^{+} Resting CD8 αβ T	CD3^{+}CD14^{-}CD19^{-}CD56^{-}TCR$_{αβ}^{+}$TCR$_{γδ}^{-}$CD4^{-}CD8^{+} CD27^{+}CD28^{+}PD-1^{+}PD-L1^{-}
PD-1^{+} B	CD3^{-}CD14^{-}CD19^{+}CD56^{-}PD-1^{+}PD-L1^{-}
PD-L1^{+} cells	
PD-L1^{+} PBMC	PD-1^{-}PD-L1^{+}
PD-L1^{+} monocyte	CD3^{-}CD14^{+}CD19^{-}CD56^{-}PD-1^{-}PD-L1^{+}
PD-L1^{+} NK	CD3^{-}CD14^{-}CD19^{-}CD56^{+}PD-1^{-}PD-L1^{+}
PD-L1^{+} NKT	CD3^{+}CD14^{-}CD19^{-}CD56^{+}PD-1^{-}PD-L1^{+}
PD-L1^{+} CD4 NKT	CD3^{+}CD14^{-}CD19^{-}CD56^{+}CD4^{+}CD8^{-}PD-1^{-}PD-L1^{+}
PD-L1^{+} CD8 NKT	CD3^{+}CD14^{-}CD19^{-}CD56^{+}CD4^{-}CD8^{+}PD-1^{-}PD-L1^{+}
PD-L1^{+} DC	CD3^{-}CD14^{-}CD19^{-}CD56^{-}CD11c^{+}PD-1^{-}PD-L1^{+}
PD-L1^{+} T	CD3^{+}CD14^{-}CD19^{-}CD56^{-}PD-1^{-}PD-L1^{+}
PD-L1^{+} CD4 T	CD3^{+}CD14^{-}CD19^{-}CD56^{-}CD4^{+}CD8^{-}PD-1^{-}PD-L1^{+}
PD-L1^{+} CD8 T	CD3^{+}CD14^{-}CD19^{-}CD56^{-}CD4^{-}CD8^{+}PD-1^{-}PD-L1^{+}
PD-L1^{+} B	CD3^{-}CD14^{-}CD19^{+}CD56^{-}PD-1^{-}PD-L1^{+}
PD1^{+}PD-L1^{+} cells	
PD-1^{+}PD-L1^{+} PBMC	PD-1^{+}PD-L1^{+}
PD-1^{+}PD-L1^{+} monocyte	CD3^{-}CD14^{+}CD19^{-}CD56^{-}PD-1^{+}PD-L1^{+}
PD-1^{+}PD-L1^{+} NK	CD3^{-}CD14^{-}CD19^{-}CD56^{+}PD-1^{+}PD-L1^{+}
PD-1^{+}PD-L1^{+} NKT	CD3^{+}CD14^{-}CD19^{-}CD56^{+}PD-1^{+}PD-L1^{+}
PD-1^{+}PD-L1^{+} CD4 NKT	CD3^{+}CD14^{-}CD19^{-}CD56^{+}CD4^{+}CD8^{-}PD-1^{+}PD-L1^{+}
PD-1^{+}PD-L1^{+} CD8 NKT	CD3^{+}CD14^{-}CD19^{-}CD56^{+}CD4^{-}CD8^{+}PD-1^{+}PD-L1^{+}
PD-1^{+}PD-L1^{+} DC	CD3^{-}CD14^{-}CD19^{-}CD56^{-}CD11c^{+}PD-1^{+}PD-L1^{+}
PD-1^{+}PD-L1^{+} T	CD3^{+}CD14^{-}CD19^{-}CD56^{-}PD-1^{+}PD-L1^{+}
PD-1^{+}PD-L1^{+} CD4 T	CD3^{+}CD14^{-}CD19^{-}CD56^{-}CD4^{+}CD8^{-}PD-1^{+}PD-L1^{+}
PD-1^{+}PD-L1^{+} CD8 T	CD3^{+}CD14^{-}CD19^{-}CD56^{-}CD4^{-}CD8^{+}PD-1^{+}PD-L1^{+}
PD-1^{+}PD-L1^{+} B	CD3^{-}CD14^{-}CD19^{+}CD56^{-}PD-1^{+}PD-L1^{+}
MHC II^{+} cells	
MHC II^{+} monocyte	CD3^{-}CD14^{+}CD19^{-}CD56^{-}MHC II^{+}
MHC II^{+} NK	CD3^{-}CD14^{-}CD19^{-}CD56^{+}MHC II^{+}
MHC II^{+} NKT	CD3^{+}CD14^{-}CD19^{-}CD56^{+}MHC II^{+}
MHC II^{+} CD4 NKT	CD3^{+}CD14^{-}CD19^{-}CD56^{+}CD4^{+}CD8^{-}MHC II^{+}
MHC II^{+} CD8 NKT	CD3^{+}CD14^{-}CD19^{-}CD56^{+}CD4^{-}CD8^{+}MHC II^{+}
MHC II^{+} DC	CD3^{-}CD14^{-}CD19^{-}CD56^{-}CD11c^{+}MHC II^{+}

MHC II^+ T	$CD3^+CD14^-CD19^-CD56^-MHC\ II^+$
MHC II^+ CD4 T	$CD3^+CD14^-CD19^-CD56^-CD4^+CD8^-MHC\ II^+$
MHC II^+ CD8 T	$CD3^+CD14^-CD19^-CD56^-CD4^-CD8^+MHC\ II^+$
MHC II^+ B	$CD3^-CD14^-CD19^+CD56^-MHC\ II^+$

DC, dendritic cell; MHC II, major histocompatibility complex class II; NK, natural killer cell; NKT, natural killer T cell; PD-1, programmed cell death 1; PD-L1, programmed cell death ligand 1; TCR, T cell receptor.
