# Supplementary material for: Novel immunoprofiling method for diagnosing SLE and evaluating therapeutic response
Source: Lupus Sci Med. 2022 Jun 22;9(1):e000693. doi: 10.1136/lupus-2022-000693 (PMC9226994; doi:10.1136/lupus-2022-000693)
Supplement: Supplementary data [file lupus-2022-000693supp002.pdf]

Supplementary Figure 1. Schematic illustration of the immune signature calculation.

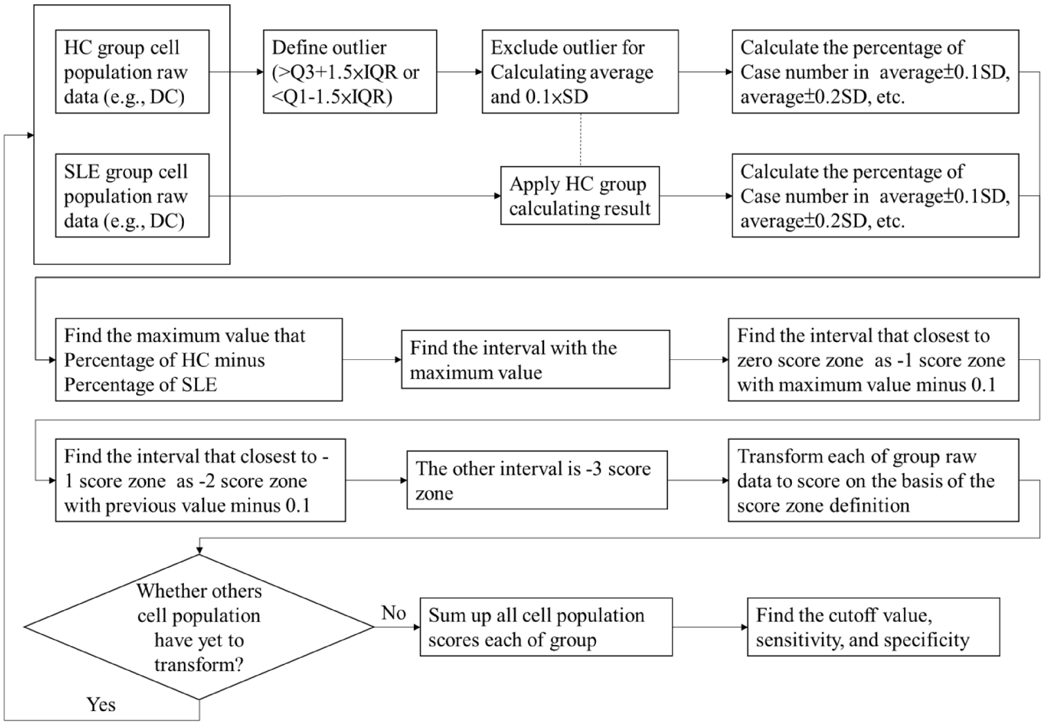

Contents of the immune subsets from the HC group were trimmed followed by the calculating average and standard deviation (SD). Then, we applied average ± 0.1×SD into the HC and the SLE groups to determine the zero zone. We further calculated zero-zone ± 0.1×SD in the HC group and used to rank the content of each subset as -1, -2, and -3. Finally, we summarized all ranked subsets as the immune signature. Abbreviation: DC, dendritic cell; HC, healthy control; IQR, interquartile range; Q1, 1st quartile; Q3, 3rd quartile.
